# Oneida County Medical Society

**Published:** 1873-03

**Authors:** 


### Correspondence.

### Oneida County Medical Society.

*To the Editor of the Buffalo Medical and Surgical Journal.*

MY DEAR DOCTOR.—In the February number of your Journal, at page 279, under the head of "Editorial," you have done great injustice to us, "Doctors" of the Oneida County Medical Society, and to the society itself. You have most bitterly censured a large, active and regular society for what two or three members attempted to do, but ignobly failed in the attempt.

In "Editorial," you say, (which assertion has not one *molecule* of truth in it) "the Oneida County Medical Society sends us a circular with resolutions, proposing to the profession" certain changes in our organization and code of ethics, which are "too absurd for discussion." (I am free to admit that no sane medical man who justly regards the honor, the integrity and usefulness of the medical profession, could for a moment dream of asking for any such change as the one proposed.)

You also say, (what would have been well said if the facts in the case had been such as you had been led to believe,) "the Governor should be asked to appoint a commission of experts, on insanity, to inquire into the mental equilibrium of Oneida County Doctors," for the purpose of keeping them on the "level."

Mr. Editor, this slander of the "Oneida County Medical Society" is wholly without excuse or paliation—and I here submit, (by way of parenthesis,) that if a commission of lunacy is to be appointed (and I think one should be) that such commission should be instructed to inquire into the *mental* and *moral* condition of those who have *coined* and *circulated* a base slander against a regular and *old school* medical society. Certainly we have neither said nor done anything which should cast a shadow upon our professional "equilibrium."

The following are the facts, and all the facts in the case.

At the semi-annual meeting of the Oneida County Medical Society, held in the city of Rome on the second Tuesday of January,

1873, Dr. E. Hutchinson of Utica, secretary of our County Medical Society, offered the resolutions which were sent to you, and I presume to many others. After discussion, said resolutions were laid upon the table by an almost unanimous vote; two or three only favoring their adoption.

I was not present at the Rome meeting of our society, but am authorized by those members who did attend that meeting, to make this statement.

The "Circular," to which you refer in your editorial, is a bogus production, calculated and designed to injure and degrade our old and steadfast society. It is no more the expression of our society than the resolutions and speech of Dr. C. B. Coventry on the same subject, presented to the State Medical Society at its annual session in February, 1872, was the expression of our State Society. (See Transactions" of 1872, pages 44, 5, 6 and 7.)

Both fell, as they should have done, still born—mere abortions.

And now, my dear doctor, in view of the facts in the case, and the errors into which some one has misled you, please make the *amende honorable* in your next issue, and thereby vindicate the Oneida County Medical Society, one of the most regular, active and "level" in the State, from the unjust charges of disloyalty, want of "mental equilibrium" and professional integrity.

ONEIDA.

———:o:———

